# The effects of lineup size on the processes underlying eyewitness decisions

**DOI:** 10.1038/s41598-023-44003-y

**Published:** 2023-10-11

**Authors:** Nicola Marie Menne, Kristina Winter, Raoul Bell, Axel Buchner

**Affiliations:** Department of Experimental Psychology, Heinrich Heine University Düsseldorf, Düsseldorf, Germany

**Keywords:** Psychology, Human behaviour

## Abstract

Here we apply the two-high threshold eyewitness identification model to identify the effects of lineup size on the detection-based and non-detection-based processes underlying eyewitness decisions. In Experiment 1, lineup size was manipulated by showing participants simultaneous or sequential lineups that contained either three or six persons. In Experiment 2, the lineups contained either two or five persons. In both experiments, the culprit was better detected in smaller than in larger lineups. Furthermore, participants made fewer guessing-based selections in smaller than in larger lineups. However, guessing-based selection in larger lineups was not increased to a level sufficient to offset the effect of increased protection of suspects in larger lineups due to the fact that the guessing-based selections that occur are distributed across more persons. The results show that increasing the lineup size causes several changes in the detection-based and non-detection-based processes underlying eyewitness decisions.

## Introduction

Eyewitness identification via lineup procedures is a major source of evidence in criminal investigations. In a lineup procedure, an eyewitness is presented with a suspect (who is guilty or innocent) along with a number of fillers (who are known to be innocent) and is asked to make an identification or to reject the lineup. However, human memory is unreliable and highly prone to error. According to the Innocence Project^1^, mistaken eyewitness identifications have played a role in 70 % of the more than 375 wrongful convictions that have been revealed by DNA analyses. While many structural and procedural aspects of lineups have been studied, including the lineup presentation format (e.g.,^2^), the instructions given to eyewitnesses (e.g.,^3^) or the characteristics of fillers (e.g.,^4^), there has been relatively little research addressing the question of how the number of fillers in a lineup affects eyewitness decisions. In addition, studies to date have mainly focused on the question as to which lineup size should be preferred by relying on overall measures of lineup quality (e.g.,^5–7^). The present study aims to complement the existing literature by providing a more detailed dissection of how lineup size affects the processes underlying eyewitness decisions in lineups. Improving our understanding of the processes underlying lineup size effects seems desirable considering the large variability in lineup size policies across jurisdictions. For instance, in the United States, a lineup most often contains five fillers^8^, the police in the United Kingdom typically use eight fillers^9^ and in Germany, the recommended lineup includes at least seven fillers^10^. Before considering the potential effects of lineup size on the latent processes underlying eyewitness decisions, it must be pointed out that it is plausible that increasing the lineup size diminishes the probability of suspect identifications simply due to the mathematical consequences of selecting among a larger group of persons. If participants randomly guess among the available options, the sampling probability of selecting the suspect among the fillers is inversely proportional to the size of the lineup. However, it is quite possible that variations in the number of persons that have to be considered in a lineup also affect the latent detection-based and non-detection-based processes underlying eyewitness decisions in addition to this sampling probability. Here we rely on the well-validated two-high threshold (2-HT) eyewitness identification model^11,12^ to disentangle the effects of lineup size on the detection-based and non-detection-based processes involved in eyewitness decisions.

Multinomial processing tree models, to which class the 2-HT eyewitness identification model belongs, are statistical models for categorical data that have been widely used to infer latent cognitive processes from observable behavior (for reviews, see^13,14^). These models form an easily accessible class of measurement models for which both excellent tutorials^15^ and easy-to-use free software for parameter estimation and statistical hypothesis tests exist^16^. Multinomial processing tree models are based on the assumption that an observable response originates from a sequence of latent processes. These sequences are visualized as branches in a processing tree. The branches consist of links and nodes. The links terminating in nodes represent probabilities with which certain cognitive processes occur. These are the parameters of the model that can be measured based on observable data.

**Figure 1:**
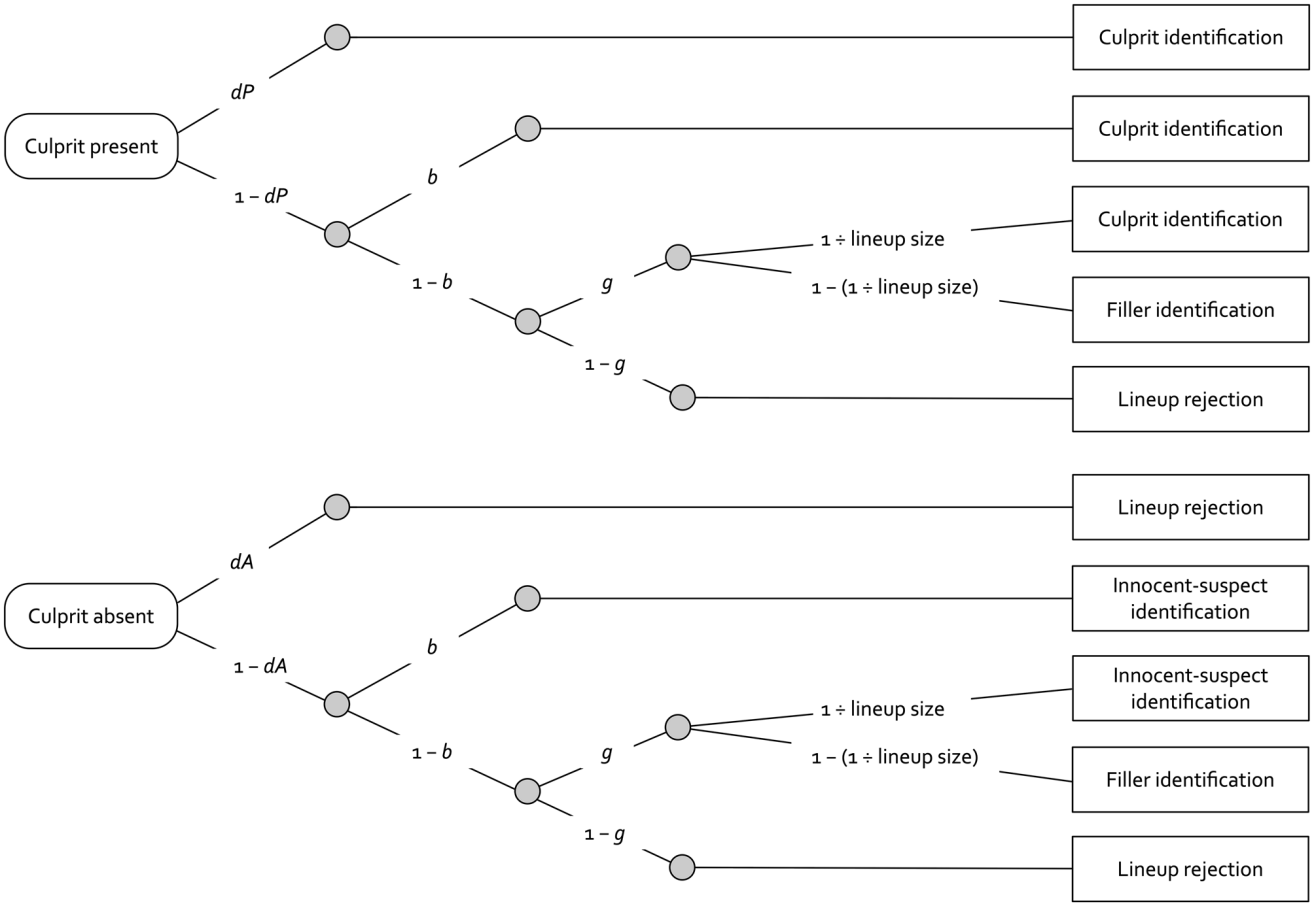
Illustration of the 2-HT eyewitness identification model. Rounded rectangles on the left side represent the two different lineup types presented at test (culprit-present and culprit-absent lineups) and the rectangles on the right side represent the observable response categories. The letters along the branches represent the probabilities of the latent processes postulated by the model (*dP*: probability of detecting the presence of the culprit; *b*: probability of biased selection of the suspect; *g*: probability of guessing-based selection among the lineup members; *dA*: probability of detecting the absence of the culprit). The random-sampling probability that is given by 1 ÷ lineup size is not a to-be-estimated parameter but a direct function of the number of persons in the lineup.

Based on the full range of data categories in a typical lineup task (i.e., suspect identifications, filler identifications and lineup rejections in both culprit-present and culprit-absent lineups), the 2-HT eyewitness identification model provides measures of the latent processes from which eyewitness decisions originate (Fig. 1). Specifically, the model serves to separately measure the detection of culprit presence (parameter *dP*), the detection of culprit absence (parameter *dA*), guessing-based selection among the lineup members (parameter *g*) and biased suspect selection in unfair lineups (parameter *b*). The model has the further advantage that it incorporates the inverse relationship between the random-sampling probability and lineup size in terms of a fixed constant that is independent of the parameters representing the detection-based and non-detection-based processes that have to be estimated from the data. In validation studies, it has been empirically shown that the parameters of the 2-HT eyewitness identification model sensitively reflect manipulations of the processes they were intended to measure in both simultaneous and sequential lineups^11,12^. The model has already been successfully applied to examine the effects of first-yes-counts instructions on guessing-based selection^17^ and to measure the effects of lineup fairness^18^.

The upper tree in Fig. 1 represents the processes that may occur in response to culprit-present lineups. The presence of the culprit is detected with probability *dP*, resulting in a correct identification of the culprit. This process is enhanced under conditions that facilitate memory formation and retrieval such as the better encoding of the culprit’s face^11,12^. If the presence of the culprit is not detected, which occurs with probability 1 − *dP*, two types of non-detection-based processes may still lead to a correct culprit identification in culprit-present lineups. With probability *b*, biased selection of the culprit may occur because the culprit stands out from the other persons in an unfair lineup. For instance, parameter *b* increases if the suspect has distinctive facial features (e.g., birthmarks, tattoos, nose piercings) that make the suspect stand out from the fillers^11,12^. No biased suspect selection occurs with the complementary probability 1 − *b*. In this case, participants may still select one of the persons in the lineup based on guessing with probability *g*. Parameter *g* increases, for instance, if the lineup instructions insinuate that the culprit is in the lineup^11,12^. Guessing-based selection leads either to the identification of the culprit with the random-sampling probability that is equal to 1 ÷ lineup size or to the identification of one of the fillers with the complementary probability 1 − (1 ÷ lineup size). Alternatively, participants may not select a lineup member based on guessing with probability 1 − *g*, in which case the lineup is falsely rejected.

The lower tree of the 2-HT eyewitness identification model represents the processes that may occur in response to culprit-absent lineups. The absence of the culprit is detected with probability *dA*, leading to a correct lineup rejection. This may occur, for instance, if all lineup members in a culprit-absent lineup can be ruled out as the culprit because their faces share a common feature (e.g., birthmarks) that the culprit did not have^12^. Culprit-absence detection fails with probability 1 − *dA*, in which case the non-detection-based processes occur in the same way as in culprit-present lineups. With probability *b*, biased selection of the innocent suspect occurs because the innocent suspect stands out from the other persons in the lineup. No biased suspect selection occurs with probability 1 − *b*. In this case, participants may still make a guessing-based selection with probability *g,* resulting in either the identification of the innocent suspect with probability 1 ÷ lineup size or the identification of a filler with probability 1 − (1 ÷ lineup size). If participants do not make a guessing-based selection, which occurs with probability 1 − *g*, then the lineup is correctly rejected. More details can be found elsewhere^11,12^.

Focusing on the role of lineup size in the model immediately clarifies the mathematical consequences of larger lineups on suspect identifications: The random-sampling probability that expresses the probability with which guessing-based selection leads to the identification of the suspect decreases with increasing lineup size. For instance, in a three-person lineup, a guessing-based selection will lead to a suspect identification with a random-sampling probability of 1/3, whereas in a six-person lineup, the random-sampling probability is reduced to 1/6.

Here we report two well-powered experiments to examine the effects of lineup size on the detection-based and non-detection-based processes underlying eyewitness decisions. Participants viewed a video of a staged crime and were subsequently presented with four lineups. In Experiment 1, lineup size was manipulated between participants by presenting lineups consisting of either three or six persons. Experiment 2 served as a conceptual replication of Experiment 1 with the only difference being that lineup size was manipulated by presenting lineups consisting of either two or five persons. In addition to simultaneous lineups (in which the lineup members are presented together), we also presented sequential lineups (in which the lineup members are presented one at a time) because sequential lineups have become the standard format for presenting lineups in countries such as the United Kingdom or Germany^10,19^.

The present study avoids two methodological problems in lineup size research that have been pointed out by Juncu and Fitzgerald^20^. First, in most studies on lineup size, no designated innocent suspect was included in culprit-absent lineups. As a consequence, the rate of innocent-suspect identifications had to be estimated by dividing the overall number of false identifications in culprit-absent lineups by the number of persons in the lineup (e.g.,^6,7^). However, this method places an upper limit on the rate of innocent-suspect identifications—the inverse of the size of the lineup—which may underestimate the real-world risk to innocent suspects because it is based on the assumption that the lineups are perfectly fair and biased selections cannot occur^20,21^. In the present experiments, culprit-absent lineups with a designated innocent suspect were used. This type of lineup composition is more ecologically valid than using culprit-absent lineups containing only fillers. In practice, the photograph of the suspect (whose guilt is unknown to the police) is often taken from a different source (e.g., from a mugshot or social media) than the photographs of the fillers (typically taken from databases) and may therefore differ to some extent from that of the fillers. Second, in some studies on lineup size, the number of persons in the lineup was confounded with the identity of the fillers such that certain fillers were only used in the larger lineups but not in the smaller lineups (for an overview, see^20^). In the present experiments, a random-selection procedure (see the Lineup procedures section below) guaranteed that the identity of the lineup fillers was not confounded with the lineup-size variable.

Among the few studies that have investigated the effects of lineup size on eyewitness decisions, some failed to find a significant difference in either the proportions of suspect identifications or the proportions of lineup rejections^22–26^. However, none of these studies had a sufficiently large sample size to detect an effect of lineup size on eyewitness decisions with a high sensitivity^27^. The results of studies using larger sample sizes indicate that there may be a trade-off between the aims of protecting the innocent suspects on the one side and of prosecuting the culprits on the other. Adding lineup fillers decreases the proportion of false innocent-suspect identifications but also decreases the proportion of culprit identifications^5–7^. However, these response rates may hide theoretically important effects as they confound different underlying processes. The increase in culprit identifications and innocent-suspect identifications in smaller compared to larger lineups may simply be a consequence of the fact that the sampling probability with which random guessing-based selection among the lineup members leads to the identification of the suspect is inversely related to lineup size. However, it is also possible to hypothesize that the increase in culprit identifications in smaller lineups may additionally be the result of enhanced culprit-presence detection. Previous research either found that lineup size did not affect the ability to discriminate between culprits and innocent suspects^6,7^ or provided evidence for an increased ability to discriminate between culprits and innocent suspects in smaller compared to larger lineups^5^. Increased culprit-presence detection in smaller lineups is expected based on the assumption that “larger lineups may prove to be more difficult due to the increase in cognitive demands required by a larger lineup, that in turn may hinder identification accuracy” (^26^, pp. 25–26). In fact, the mere processing of visual information has already been demonstrated to interfere with the accuracy of memories (e.g.,^28–30^). In the case of an increased number of filler faces in larger lineups, the additional faces represent more visual information that may require additional processing resources which may then be diverted from the to-be-detected culprit’s face, resulting in a diminished ability to detect the culprit (cf.^28–30^). Here we use the 2-HT eyewitness identification model that represents the inverse relationship between the random-sampling probability and lineup size by a fixed constant—given by 1 ÷ lineup size—which is independent of the parameters representing the detection-based and non-detection-based processes that have to be estimated from the data. Based on the literature available to date, it remains an open question as to whether culprit-presence detection, represented by the model’s parameter *dP,* is increased in smaller compared to larger lineups or whether *dP* is independent of lineup size.

The second test of interest refers to guessing-based selection. Even though both culprit identifications and innocent-suspect identifications decrease with increasing lineup size—which may at first glance be taken as evidence for a decreased reliance on guessing—, a closer look at the other data categories that are available from the lineups reveals that lineup rejections decrease and filler identifications increase with increasing lineup size^20^. This pattern of results may be taken to suggest that the protection of suspects that is granted by large lineup sizes is only caused by the dispersion of guessing-based selections among the lineup members due to the inverse relationship to the random-sampling probability while the probability with which eyewitnesses make guessing-based selections may actually increase in larger lineups compared to smaller lineups. To test this hypothesis more directly, the model’s guessing-based-selection parameter *g* was compared between the larger and the smaller lineups.

## Experiment 1

### Methods

#### Participants

Data were collected using the research panel of respondi AG based in Cologne, Germany (https://www.respondi.com) who compensated participants for their participation. Of the 1821 participants who initially filled out the socio-demographic questionnaire at the beginning of the experiment, 284 had to be excluded because they either failed to complete the experiment or withdrew the consent to use their data (*n* = 234), saw the staged-crime video more than once due to repeated participation (*n* = 39) or failed the attention check (*n* = 11; for an explanation, see the Materials and procedure section). The final sample, characterized by a diverse level of education, included the data of 1537 participants (668 female, 865 male, 4 diverse) aged between 18 and 88 years (*M* = 46, *SD* = 16). A sensitivity analysis with G*Power^31^ showed that, given a sample size of *N* = 1537, four eyewitness decisions per participant and α = β = 0.05, it was possible to detect even small effects of lineup size of size *w* = 0.05 on the model parameters across the lineup format conditions (*df* = 2). Participants were randomly assigned to one of the four lineup conditions: the three-person sequential lineup condition (*n* = 382), the six-person sequential lineup condition (*n* = 393), the three-person simultaneous lineup condition (*n* = 380) and the six-person simultaneous lineup condition (*n* = 382).

#### Ethics statement

In Experiments 1 and 2, participants gave informed consent prior to participation. The ethics committee of the Faculty of Mathematics and Natural Sciences at Heinrich Heine University granted approval for a series of experiments to which the present experiments belong. Both experiments were conducted in accordance with the Declaration of Helsinki. Participants were informed that they would see a video including elements of verbal and physical abuse. In case of any discomfort arising from watching such a video, participants were advised to withdraw from the study. At the end of the experiments, participants were debriefed that the crime had been staged for research purposes.

#### Materials and procedure

Materials and procedure were essentially as described by Winter et al.^12,17^ and Menne et al.^18^ with the exception of the lineup size manipulation described below. The experiment was conducted online. It was programmed with *Sosci Survey*^32^ and was made available via https://www.soscisurvey.de. Participants were only allowed to participate with a desktop or laptop computer, not with a tablet or smartphone.

##### Staged-crime videos

Participants were shown one of two staged-crime videos (henceforth referred to as Video 1 and Video 2). In both videos, four hooligans of the German soccer club FC Bayern München (the culprits) attacked a fan of the rivaling soccer club Borussia Dortmund (the victim) at a bus station. The victim and the culprits wore fan clothing (caps, shirts and scarfs) showing their club’s color and logo. The culprits insulted the victim, made fun of him and tossed his personal belongings around. As the video progressed, the culprits became more physically aggressive. They pushed the victim around until he got knocked to the ground. The four culprits carried on with their verbal and physical abuse of the victim until they suddenly became aware of the presence of another person approaching (not depicted in the videos). At that point, they swiftly ran away. Following the lead of other researchers (e.g.,^33–36^), our participants were shown a crime video including four culprits to increase the efficiency of data collection while maintaining ecological validity given that a substantial number of real-world crimes involve multiple culprits^37–39^.

Both videos depicted the identical crime event, following the same sequence and timing, but the victims and the culprits were different actors. However, the actors were selected in such a way that the victim of Video 1 resembled the victim of Video 2 and that each of the four culprits of Video 1 had a high resemblance (as determined by the authors) to one of the four culprits of Video 2 in terms of body shape, hair color and hairstyle (i.e., Culprit A in Video 1 resembled Culprit A in Video 2, Culprit B in Video 1 resembled Culprit B in Video 2 and so on). Participants were randomly assigned to one of the two versions of the staged-crime video. The videos lasted about 130 s and were presented at a resolution of 885 × 500 pixels. The videos allowed participants to have a clear view on the culprits’ faces.

Participants started the video by pressing the ‘Start’ button. They could not fast-forward, replay or pause the video and could only proceed to the next page after they had seen the whole video. To ensure that the video had been attended, a 10-alternatives attention-check question was displayed afterwards, asking participants to select the option that correctly indicated what type of persons had been shown in the video (correct response: “soccer fans”). Participants who failed the attention check were excluded from further analyses.

##### Lineup procedures

Immediately after the attention-check question, participants were informed that they would see several lineups with a series of faces and that their task was to identify the FC Bayern München hooligans from the video. Participants were also told that the lineups may or may not contain a previously seen face to emphasize not only the need to identify the culprit when the culprit is present but also the need to reject the lineup when the culprit is absent.

Participants were then presented with four separate lineups in a randomized order, each corresponding to one of the four culprits in the video. Depending on the lineup size condition, the lineups consisted of the facial photographs of one suspect and of either two or five fillers. Two of the four lineups were culprit-present lineups, the other two were culprit-absent lineups. The crossed lineup procedure^12,18^ was used to manipulate culprit presence. Two of the culprits of the video participants had seen were randomly selected to be presented in the two culprit-present lineups alongside the fillers. In the two culprit-absent lineups, the photographs of the suspects were photographs of the actors from the video participants had not seen. For instance, if participants had viewed Video 1, then two randomly selected culprits from Video 1 (e.g., Culprit B and Culprit D) served as the culprits in the culprit-present lineups and two culprits from Video 2 (in this example, Culprit A and Culprit C) served as the innocent suspects in the culprit-absent lineups. This lineup construction procedure helps to ensure that the photographs of the culprits and of the innocent suspects differs to the same degree from the photographs of the fillers. This represents an ecological valid procedure. In the real world, the photograph of the suspect (whose status of being innocent or guilty is unknown to the police) is often taken from a different source (e.g., from a mugshot or social media) than the photographs of the fillers, which are typically obtained from face databases.

For each lineup, five male filler faces aged between 18 and 29 years were taken from the Center for Vital Longevity Face Database^40^. Fillers were chosen based on their resemblance (as determined by the authors) to the culprits in terms of body shape, hair color and hairstyle. In the three-person lineup conditions, two fillers were randomly selected for each participant from the pool of five fillers of each lineup. The randomization ensured that the critical difference between the lineup size conditions was the number of fillers and not the filler’s identity. The positions of the suspect and filler photographs were randomized in each lineup. The photographs of the suspects and the fillers showed a front view of face and neck against a black background. All faces had neutral facial expressions. All photographs were matched for lighting and face size and were displayed at a resolution of 142 × 214 pixels. Examples of the lineups used in Experiments 1 and 2 can be found at https://osf.io/ckdbr/.

Depending on the lineup format condition, the lineups were presented either simultaneously or sequentially. In simultaneous lineups, the photographs of the suspect and the fillers were shown together in one row. Participants could identify a person as the culprit by pressing the “Yes, was present” button underneath the photograph of this person or they could reject the lineup by pressing the “No, none of these persons was present” button located to the right of each lineup. After their decision, participants were asked to indicate their confidence in their response to make the procedure similar to that of a real police lineup. The participants could then initiate the presentation of the subsequent lineup by pressing the “Next” button. In sequential lineups, the photographs of the suspect and the fillers were presented, one at a time, in random succession. For each lineup member, participants decided whether or not the depicted person was one of the culprits of the video they had seen by pressing the “Yes, was present” button underneath the photograph or the “No, this person was not present” button located at the right side of the photograph. Participants were required to make a decision before they could proceed to the next photograph. After making each decision, participants were asked to indicate their confidence in their response. If participants identified more than one face in a lineup, we followed the standard police procedure in Germany and the United States^10,41,42^, considering the last identification as a revision of any prior identification. Therefore, the last identification was used in the present analysis. If participants identified none of the persons in the lineup as one of the culprits, the lineup was counted as rejected. After participants had made their decision in the fourth lineup, they were debriefed and thanked for their time.

### Results

For all analyses reported in this article, parameter estimates and goodness-of-fit tests were calculated using *multiTree*^16^. The α level was set to 0.05. The observed response frequencies and proportions for Experiments 1 and 2 are shown in Table 1.

**Table 1:** Response frequencies and proportions (in parentheses) as a function of lineup format and lineup size observed in Experiments 1 and 2.

Lineup format	Lineup size	Culprit-present lineups			Culprit-absent lineups		
		Culprit identifications	Filler identifications	Lineup rejections	Innocent-suspect identifications	Filler identifications	Lineup rejections
Experiment 1							
Sequential	Three persons	386 (0.51)	205 (0.27)	173 (0.23)	206 (0.27)	241 (0.32)	317 (0.41)
	Six persons	273 (0.35)	339 (0.43)	174 (0.22)	127 (0.16)	374 (0.48)	285 (0.36)
Simultaneous	Three persons	371 (0.49)	120 (0.16)	269 (0.35)	139 (0.18)	184 (0.24)	437 (0.58)
	Six persons	283 (0.37)	191 (0.25)	290 (0.38)	87 (0.11)	249 (0.33)	428 (0.56)
Experiment 2							
Sequential	Two persons	499 (0.61)	124 (0.15)	193 (0.24)	259 (0.32)	161 (0.20)	396 (0.49)
	Five persons	320 (0.40)	316 (0.39)	166 (0.21)	143 (0.18)	378 (0.47)	281 (0.35)
Simultaneous	Two persons	433 (0.55)	72 (0.09)	281 (0.36)	204 (0.26)	98 (0.12)	484 (0.62)
	Five persons	320 (0.41)	172 (0.22)	296 (0.38)	115 (0.15)	232 (0.29)	441 (0.56)

The proportions are rounded to two decimal places and thus do not always add up to 1.

**Figure 2:**
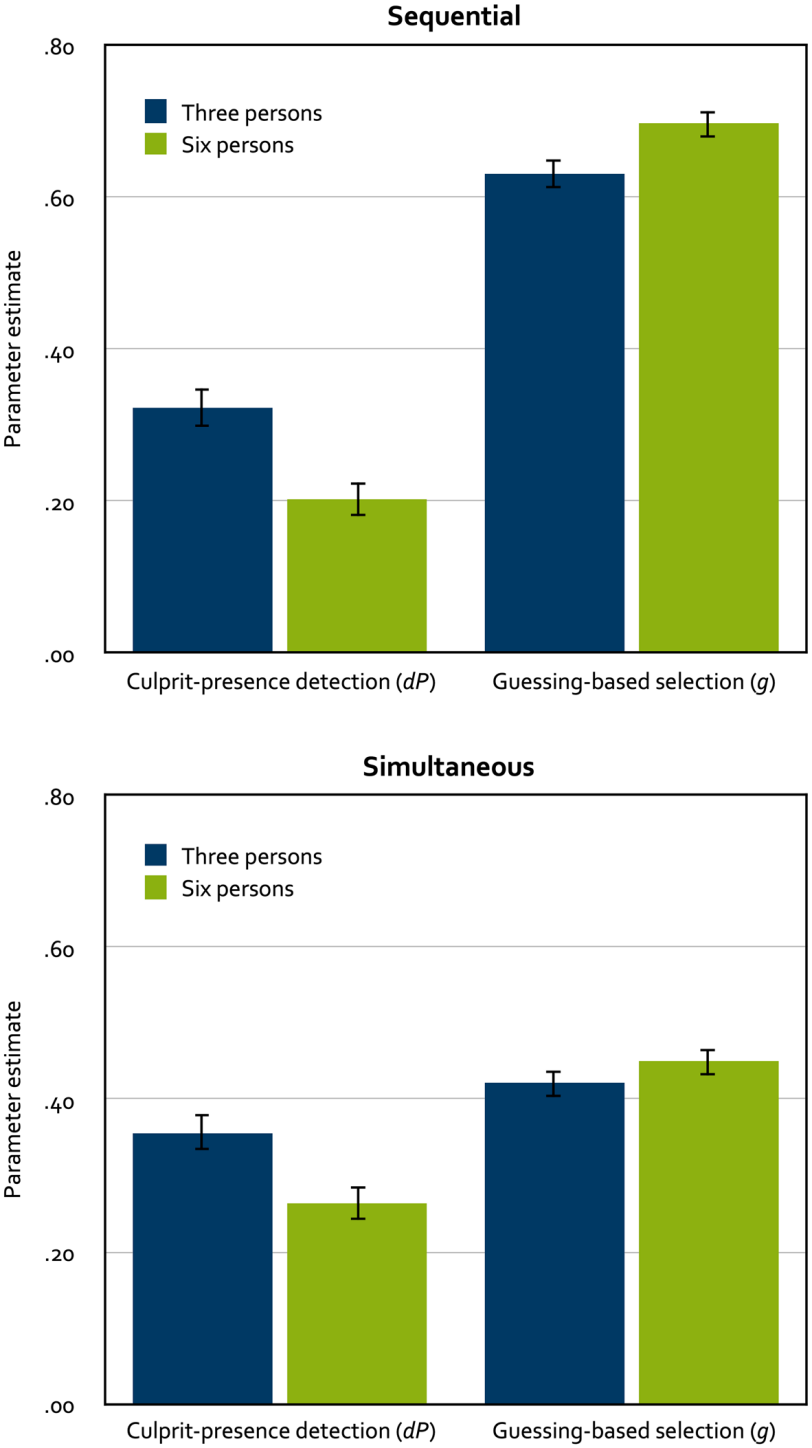
Parameter estimates of parameter *dP* reflecting the probability of detecting the presence of the culprit (left panels) and parameter *g* reflecting the probability of guessing-based selection among the lineup members (right panels) as a function of lineup size for sequential lineups (top panels) and simultaneous lineups (bottom panels) in Experiment 1. The error bars represent standard errors.

To analyze the results of Experiment 1, we needed four instances of the model illustrated in Fig. 1, one for the three-person sequential lineups, one for the six-person sequential lineups, one for the three-person simultaneous lineups and one for the six-person simultaneous lineups. In the three-person lineup conditions, the constant 1 ÷ lineup size was set to 0.33333 to approximate the random-sampling probability of 1/3. In the six-person lineup conditions, 1 ÷ lineup size was set to 0.16667 to approximate the random-sampling probability of 1/6. Our aim was to use a base model that was as simple as possible. Therefore, we imposed parameter restrictions onto the 2-HT eyewitness identification model that were the same as those used in prior applications of this model^11,12,17^. First, given that the same sets of suspects and fillers were used in all conditions, lineup fairness must necessarily be the same across the four conditions. Therefore, the biased-suspect-selection parameter *b* was set to be equal across the four lineup conditions. Second, there was no a priori reason to assume that the ability to detect the absence of the culprit should differ among the conditions (for manipulations that can be expected to influence the ability to detect the absence of the culprit, see^11,12^). Therefore, the culprit-absence-detection parameter *dA* was set to be equal across the four conditions. The base model incorporating these restrictions fit the data, *G*^*2*^(6) = 8.39, *p* = 0.211. The estimates of parameters *b* and *dA* were 0.07 (*SE* = 0.01) and 0.11 (*SE* = 0.02), respectively. The estimates of parameters *dP* and *g* are displayed in Fig. 2.

One advantage of multinomial processing tree models is that hypothesis tests can easily be performed directly at the level of the postulated processes (for details, see the tutorial by^15^). For instance, the hypothesis that culprit-presence detection differs between the three-person lineups and the six-person lineups can be tested by setting the culprit-presence-detection parameter *dP* to be equal between these conditions. If the fit of the model including this restriction is significantly worse than the fit of the base model not including this restriction, then it can be concluded that culprit-presence detection differs between conditions in the direction indicated by the parameter estimates. Participants in the three-person lineup conditions were significantly more likely to detect the culprit than participants in the six-person lineup conditions, ∆*G*^*2*^(2) = 22.17, *p* < 0.001. In addition, the probability of guessing-based selection was significantly higher when the lineups contained six persons than when the lineups contained three persons, ∆*G*^*2*^(2) = 10.86, *p* = 0.004.

### Discussion

The results of Experiment 1 support the hypothesis that culprit-presence detection is better in smaller compared to larger lineups. Participants were more likely to identify the culprit in three-person lineups than in six-person lineups. In addition, the model-based analysis demonstrated that participants were more likely to select a lineup member based on guessing in six-person lineups than in three-person lineups, thus providing direct evidence that increasing the lineup size causes increased guessing-based selection. The consequences of these guessing-based selections in terms of whether the suspect or a filler is selected is then given by the random-sampling probability, which is a fixed constant in the analysis.

Given the debate about the need for replication^43^, it seemed desirable to test the reliability and robustness of these findings before drawing firm conclusions. Therefore, Experiment 2 served as a conceptual replication of Experiment 1, the only difference to Experiment 1 being that participants were presented with lineups consisting of either two or five persons.

## Experiment 2

### Method

#### Participants

Participants were recruited and compensated as in Experiment 1. Of the 1851 participants who initially filled out the socio-demographic questionnaire at the beginning of the experiment, 255 had to be excluded because they either failed to complete the experiment or withdrew the consent to use their data (*n* = 217), saw the staged-crime video more than once due to repeated participation (*n* = 19) or failed the attention check (*n* = 19). The final sample, characterized by a diverse level of education, included the data of 1596 participants (717 female, 872 male, 7 diverse) aged between 18 and 74 years (*M* = 46, *SD* = 16). A sensitivity analysis with G*Power^31^ showed that, given a sample size of *N* = 1596, four eyewitness decisions per participant and α = β = 0.05, it was possible to detect even small effects of lineup size of size *w* = 0.05 on the model parameters across the lineup format conditions (*df* = 2). Participants were randomly assigned to one of the four lineup conditions: the two-person sequential lineup condition (*n* = 408), the five-person sequential lineup condition (*n* = 401), the two-person simultaneous lineup condition (*n* = 393) and the five-person simultaneous lineup condition (*n* = 394).

#### Materials and procedure

The materials and procedure were the same as those used in Experiment 1 with the following exception. Instead of presenting three-person or six-person lineups, the participants were randomly assigned to view two-person or five-person lineups consisting of the facial photographs of the suspect and either one or four fillers, respectively. Parallel to Experiment 1, the fillers were randomly selected from the pool of five fillers of each lineup.

### Results

**Figure 3:**
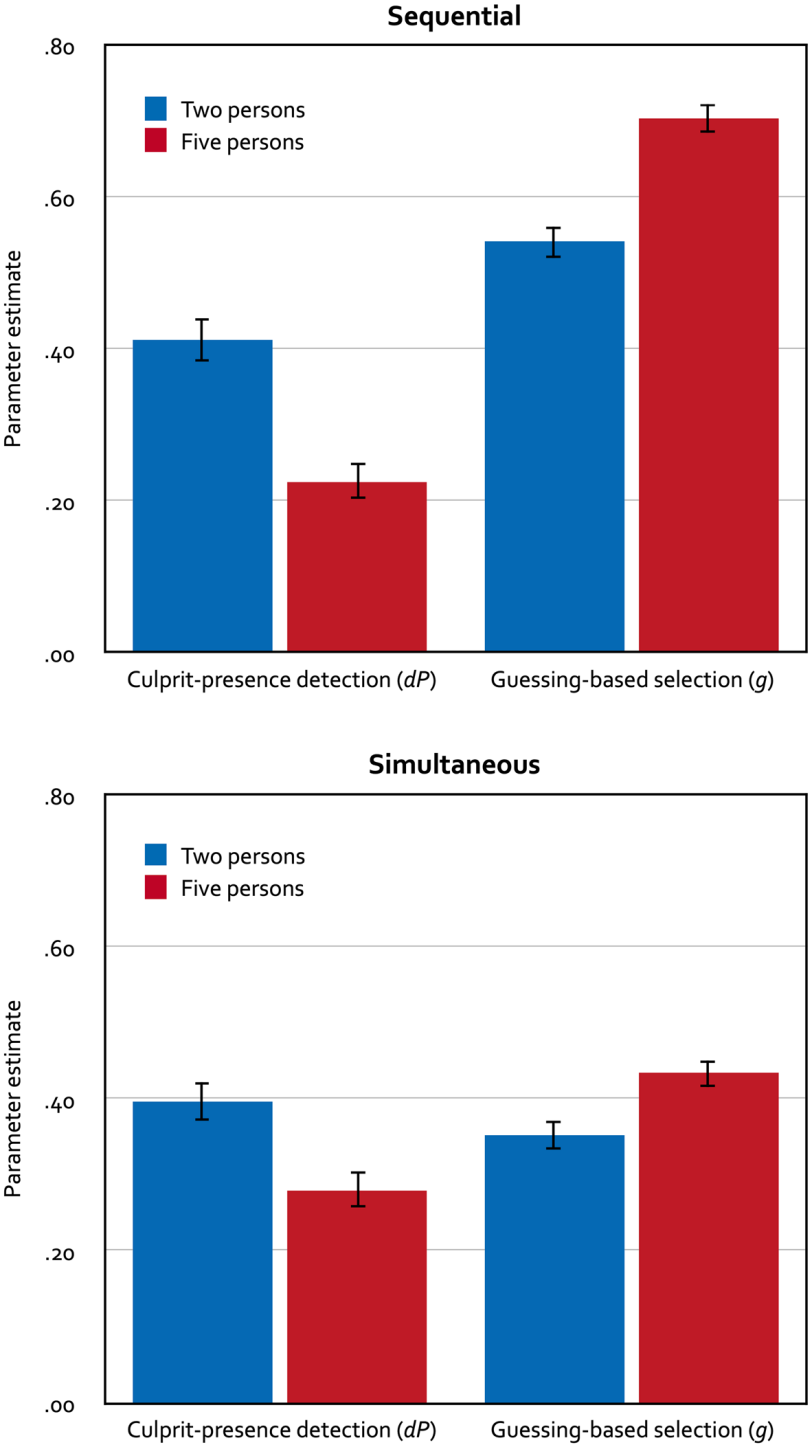
Parameter estimates of parameter *dP* reflecting the probability of detecting the presence of the culprit (left panels) and parameter *g* reflecting the probability of guessing-based selection among the lineup members (right panels) as a function of lineup size for sequential lineups (top panels) and simultaneous lineups (bottom panels) in Experiment 2. The error bars represent standard errors.

To analyze the results of Experiment 2, we needed four instances of the model depicted in Fig. 1, one for the two-person sequential lineups, one for the five-person sequential lineups, one for the two-person simultaneous lineups and one for the five-person simultaneous lineups. In the two-person lineup conditions, the random-sampling probability is given by 1 ÷ lineup size and was thus set to 0.5. In the five-person lineup conditions, the random-sampling probability is given by 1 ÷ lineup size and was thus set to 0.2. The same assumptions as in Experiment 1 were used to arrive at the base model. Specifically, the biased-suspect-selection parameter *b* and the culprit-absence-detection parameter *dA* were each set to be equal across the four lineup conditions. The model incorporating these restrictions fit the data, *G*^*2*^(6) = 12.14, *p* = 0.059. The estimates of parameters *b* and *dA* were 0.10 (*SE* = 0.01) and 0.11 (*SE* = 0.02), respectively. The estimates of parameters *dP* and *g* are displayed in Fig. 3.

Participants in the two-person lineup conditions were significantly more likely to detect the culprit than participants in the five-person lineup conditions, ∆*G*^*2*^(2) = 38.72, *p* < 0.001. In addition, the probability of guessing-based selection was significantly higher when the lineups contained five persons than when the lineups contained two persons, ∆*G*^*2*^(2) = 62.98, *p* < 0.001.

### Discussion

The aim of Experiment 2 was to determine whether the main findings of Experiment 1 could be replicated using lineups consisting of either two or five persons. As in Experiment 1, culprit-presence detection was better in smaller compared to larger lineups and increasing the lineup size caused increased guessing-based selection among the lineup members, thus supporting the robustness of those findings.

## General discussion

In many jurisdictions, larger minimum lineup sizes are recommended^20,44^ presumably because large lineups seem to be better than small lineups at protecting the suspect from being selected based on guessing. This protective effect may already result from the decreased sampling probability when eyewitnesses randomly select a person from a larger lineup. However, it is unclear whether having to consider a larger number of faces in a lineup may have additional effects on detection-based and non-detection-based processes. The aim of the present study was to apply the 2-HT eyewitness identification model to identify the effects of lineup size on the latent processes underlying eyewitness decisions. In Experiment 1, participants were presented with simultaneous or sequential lineups consisting of either three or six persons. In Experiment 2, participants were presented with simultaneous or sequential lineups consisting of either two or five persons. Taken together, the data of Experiments 1 and 2 provide a coherent picture by showing a decrease of culprit-presence detection and an increase in guessing-based selections with increasing lineup size.

The model-based results thus demonstrate that the increased culprit-identification rates in the smaller compared to the larger lineups, as also previously found in other studies on lineup size^5–7^, cannot solely be attributed to the increased sampling probability with which random guessing-based selection among the lineup members leads to suspect identifications in smaller lineups. It is also a consequence of improved culprit-presence detection in smaller compared to larger lineups. Here we can only speculate as to why this is the case. It is well established that the processing of visual information can interfere with memory of visual details (e.g.,^28–30^). With an increasing number of fillers in a lineup, a larger portion of resources may be devoted to the processing of the visual details of more and more fillers’ faces, leaving fewer and fewer resources for processing the visual details of the suspect’s face, resulting in a diminished ability to detect the culprit. Whatever the reason, the higher probability of culprit-presence detection is an interesting feature of smaller compared to larger lineups. As a side note, in the present experiments participants saw not one but four lineups and were thus exposed to identification conditions which may perhaps have been even more resource-demanding than the identification conditions in studies with only one lineup. However, the effect of lineup size on culprit-presence detection is clearly present even when only the data of the first of the four lineups are analyzed. This is the case for the data from both Experiments 1 and 2 (the data and equation files of these analyses are available at https://osf.io/ckdbr/). This suggests that the effect of lineup size is robust regardless of whether one or more lineups are used.

An interesting implication of the present analysis is that the effects of lineup size on the non-detection-based processes can be dissected into different components. Larger lineups in comparison to smaller lineups drive down the probability with which the suspect is selected given that guessing-based selection occurs (i.e., 1 ÷ lineup size, see Fig. 1). This property of larger lineups is quite obvious. Analogously, when a lecturer prepares an exam with multiple-alternative forced-choice items, more instead of fewer response options seem desirable so that students have a lower probability of selecting the correct option when making guessing-based selections. To illustrate this point using the present Experiment 1, if eyewitnesses select a person from the lineup based on guessing, which occurs with the conditional probability *g*, then the conditional probability with which the suspect is sampled is 1/3 in a three-person lineup, but only 1/6 in a six-person lineup. The 2-HT eyewitness identification model represents this simple mathematical consequence of sampling from more options explicitly in the sampling probability given by the constant 1 ÷ lineup size. The probability with which the suspect is selected based on guessing is *g* · (1 ÷ lineup size). To illustrate, the probability with which the suspect is selected based on guessing is *g* · 1/3 in a three-person lineup and *g* · 1/6 in a six-person lineup. However, including more fillers also affects the probability of guessing-based selection, represented by the model parameter *g*. In a lineup, each face provides an opportunity for a guessing-based selection. Larger lineups provide more opportunities for guessing-based selections than smaller lineups. For instance, in a three-person lineup, the eyewitness has three opportunities to make a guessing-based selection, whereas in a six-person lineup, the eyewitness has six opportunities to make a guessing-based selection. It is thus plausible that the overall probability of making a guessing-based selection in a lineup (parameter *g*) increases with increasing lineup size, which is indeed what was found in the present model-based analysis. Just as in the eyewitness situation, including a larger number of plausible response options in multiple-alternative forced-choice questions provides more opportunities for guessing a false option and, as a result, impairs performance by increasing the number of false answers and by decreasing the number of correct answers (e.g.,^45,46^). Similarly, at the level of raw response rates, larger lineups in the present experiments were associated with an increased rate of filler identifications and a decreased rate of rejections of culprit-absent lineups (see Table 1). This result is in line with the findings of Juncu and Fitzgerald^20^ who meta-analytically reviewed the body of lineup size research. They found an increase in filler identifications and a decrease in lineup rejections when lineup size increased, which already suggest at the level of raw response rates that increasing the lineup size enhances guessing-based selection.

To summarize, larger lineups provide more opportunities for guessing-based selection than smaller lineups, as a consequence of which guessing-based selections become more likely (parameter *g* is larger for larger lineups than for smaller lineups). However, if guessing-based selection occurs, then the selections are dispersed over a larger number of persons, including fillers that are known to be innocent (the sampling probability given by the constant 1 ÷ lineup size is smaller for larger lineups than for smaller lineups). Given that the probability with which the suspect is selected based on guessing is given by *g* · (1 ÷ lineup size), the question is whether the increase in guessing-based selection, as represented by parameter *g*, is compensated or even overcompensated by a decrease of the sampling probability given by the constant 1 ÷ lineup size. In both Experiments 1 and 2, larger lineups were associated with increased guessing-based selection compared to smaller lineups, but the decrease in the sampling probability in larger lineups overcompensated this effect. As a result, larger lineups were associated with a decreased rate of suspect identifications at the level of the raw response rates in both experiments (see Table 1). The increased protection of the suspect that is seen as the main advantage of larger lineups compared to smaller lineups was thus replicated in the present experiments. This result is consistent with findings of previous research on lineup size (e.g.,^5–7,20^).

In contrast to most prior work, we included both simultaneous and sequential lineups in the present experiments. Given that in some countries such as the United Kingdom or Germany, the sequential format is the standard way for presenting police lineups^10,19^, it is important to investigate the effects of lineup size in both lineup formats. The present results suggest that increasing the size of the lineups decreases culprit-presence detection and increases guessing-based selection in both simultaneous and sequential lineups. Moving beyond the main research question, it seems noteworthy that culprit-presence detection did not differ significantly between the simultaneous and the sequential lineup conditions in both experiments (Experiment 1: ∆*G*^*2*^(2) = 5.47, *p* = 0.065; Experiment 2, ∆*G*^*2*^(2) = 3.24, *p* = 0.198) although culprit-presence detection was descriptively better for simultaneous than for sequential lineups in all conditions except in the two-person lineup condition in Experiment 2 (see Figs. 2 and 3). It may seem more surprising, at least at first glance, that guessing-based selection was significantly more likely in the sequential lineup conditions than in the simultaneous lineup conditions in both Experiment 1, ∆*G*^*2*^(2) = 209.42, *p* < 0.001, and Experiment 2, ∆*G*^*2*^(2) = 210.16, *p* < 0.001. This finding is in line with previous model-based analyses^12,18^ but contradicts the common assumption that sequential lineups induce more conservative responding than simultaneous lineups^47^. Here it seems relevant that, in contrast to most previous research, participants in the sequential lineup conditions in the present experiments were not informed that only their first positive response would count. Instead, the presentation of the sequential lineups continued after a positive identification had been made; only the participant’s last identification was counted as an identification decision (if there was no identification decision, then the lineup was counted as rejected). This sequential lineup protocol was used here because it corresponds to standard police protocols^9,10,41,48^ and to the original sequential lineup protocol outlined by Lindsay and Wells^49^. Horry et al.^42^ have demonstrated that a lineup is more likely to be rejected and the suspect is less likely to be selected when a first-yes-counts protocol is used, strongly suggesting that a first-yes-counts protocol discourages participants from guessing. This finding is to be expected because participants will be reluctant to use their only positive response too early in the sequence, not knowing whether better alternatives might be presented later in the sequence. The more ecologically valid sequential lineup protocol applied here gives participants the opportunity to change their mind by allowing them to select a lineup member later in the sequence even though another lineup member has already been selected earlier. In fact, there is direct evidence that the probability of guessing-based selection is significantly less likely when first-yes-counts instructions are used than when the standard police protocol is used^17^.

## Conclusion

The purpose of the present study is to contribute to a better understanding of lineup size effects on eyewitness decisions using the 2-HT eyewitness identification model. By taking into account the full range of data categories observed in typical lineup procedures (i.e., suspect identifications, filler identifications and lineup rejections in both culprit-present and culprit-absent lineups), the model provides new insights into how lineup size affects the latent detection-based and non-detection-based processes underlying eyewitness decisions. The results demonstrate that, compared to smaller lineups, larger lineups are associated with a decreased ability to detect the culprit in the lineup and an increased probability of selecting a lineup member based on guessing. However, the increase in guessing-based selection in larger lineups is overcompensated by a reduced probability of randomly selecting the suspect among the lineup members if guessing-based selection occurs. As a consequence, the rate of innocent-suspect identifications decreases with increasing lineup size, albeit to a lesser degree than the rate of culprit identifications. In line with previous research^20^, the present results indicate that there is a trade-off between the aims of providing optimal conditions for detecting the culprit on the one side and protecting the suspect from the consequences of guessing-based selections on the other. Specifically, under the circumstances realized in the present experiments, larger lineups were associated with a disadvantage in culprit-presence detection and guessing-based selection while their main advantage may be seen in a broader dispersion of those guessing-based selections among the lineup members.

## Data Availability

The data and equation files of all analyses have been made publicly available at the Open Science Framework and can be accessed at https://osf.io/ckdbr/.
